# Hypothesis and Theory: Revisiting Views on the Co-evolution of the Melanocortin Receptors and the Accessory Proteins, MRAP1 and MRAP2

**DOI:** 10.3389/fendo.2016.00079

**Published:** 2016-06-28

**Authors:** Robert M. Dores

**Affiliations:** ^1^Department of Biological Sciences, University of Denver, Denver, CO, USA

**Keywords:** melanocortin receptors, MRAP1, MRAP2, MC2R, MC5R, evolution

## Abstract

The evolution of the melanocortin receptors (MCRs) is closely associated with the evolution of the melanocortin-2 receptor accessory proteins (MRAPs). Recent annotation of the elephant shark genome project revealed the sequence of a putative MRAP1 ortholog. The presence of this sequence in the genome of a cartilaginous fish raises the possibility that the *mrap1* and *mrap2* genes in the genomes of gnathostome vertebrates were the result of the chordate 2R genome duplication event. The presence of a putative MRAP1 ortholog in a cartilaginous fish genome is perplexing. Recent studies on melanocortin-2 receptor (MC2R) in the genomes of the elephant shark and the Japanese stingray indicate that these MC2R orthologs can be functionally expressed in CHO cells without co-expression of an exogenous *mrap1* cDNA. The novel ligand selectivity of these cartilaginous fish MC2R orthologs is discussed. Finally, the origin of the *mc2r* and *mc5r* genes is reevaluated. The distinctive primary sequence conservation of MC2R and MC5R is discussed in light of the physiological roles of these two MCR paralogs.

## Introduction

In many respects, the features of the melanocortin receptor (MCR) gene family (i.e., *mc1r, mc2r, mc3r, mc4r, mc5r*) are rather straightforward. These G Protein-coupled receptors are only found in chordates (1), and the proliferation of paralogous genes in this family has been influenced by the two genome duplication events that occurred during the early evolution of the chordates (2–4). In addition, these receptors appear to be predominately coupled to a cAMP/PKA pathway at their respective target cells (5). Finally, all of the MCRs are activated by one or more of the melanocortin-related peptides (i.e., ACTH, α-MSH, β-MSH, γ-MSH, or δ-MSH), which are derived from the precursor protein, POMC in gnathostomes (6), and the precursors POM or POC in lampreys (7).

There are also features of this gene family that are somewhat unique. For example, some of the MCRs interact with the accessory proteins melanocortin-2 receptor accessory protein (MRAP)1 and MRAP2 (8, 9), and these interactions can affect receptor trafficking and activation. In addition, for teleosts and tetrapods, the MC2R paralog has exclusive ligand selectivity for ACTH as compared to the more permissive ligand selectivity of the other MCR paralogs for ACTH and the MSH-sized ligands (10). Finally, while it is assumed that two genome duplications should yield four paralogous genes, there are five paralogous genes present in this family. Hence, the origin of the fifth gene and the physiological significance of the fifth gene are another issue that will be revisited.

## Phylogeny and Proposed Evolution of the MRAPs

Following the initial cloning of the five MCRs, pharmacology studies for each receptor were done in heterologous non-adrenal cortex-derived mammalian cell lines with one exception – MC2R (6). Mountjoy et al. (11) found that in order to examine the ligand selectivity of human MC2R, the receptor cDNA needed to be expressed in Cloudman S91 melanoma cells; a cell line that endogenously expresses the *Mc1r* gene. Subsequent studies would show that mammalian MC2R orthologs could be functionally expressed in cell lines derived from adrenal cortex cells, but not in non-adrenal mammalian cell lines (12–15). These observations contributed to the discovery of the accessory protein, MRAP (16).

Melanocortin-2 receptor accessory protein is a single chain polypeptide with one membrane-spanning domain. This transmembrane (TM) protein forms a homodimer at the endoplasmic reticulum in which the two monomers are oriented in an anti-parallel manner [reverse topology; for reviews, see Ref. (8, 9)]. In the human genome, there are two paralogous *MRAP* genes, *MRAP* or *MRAP1* (16), and *MRAP2* (17). For this discussion, “*mrap*” will be used to refer to the ancestral accessory protein gene, and *mrap1* and *mrap2* will be used to designate the two paralogous members of the gene family. As a reference for the discussion that will follow, Table 1 summarizes the observations from Chan et al. (17) with respect to the effects of human MRAP1α and human MRAP2 on the activation and trafficking of the five human MCRs.

**Table 1:** **Summary of the interactions between human melanocortin receptors and human MRAP1α and human MRAP2**.

	MRAP1α		MRAP2	
	Trafficking	Activation	Trafficking	Activation
MC1R	Not required	Not required	Not required	Lowers
MC2R	Facilitates	Required	Facilitates	Required
MC3R	Not required	Lowers	Not required	Lowers
MC4R	Restricts	Lowers	Restricts	Lowers
MC5R	Restricts	Lowers	Restricts	Lowers

*Chan et al. (17) expressed individual human melanocortin receptors in CHO cells either in the presence or absence of either human MRAP1α or human MRAP2, and measured either trafficking to the plasma membrane or activation with human ACTH (1–39) (MC2R; single dose 10^−6^M) or NDP-MSH (MC1R, MC3R, MC4R, MC5R: single dose 10^−9^M). For the trafficking experiments, “not required” indicates that co-expression with an MRAP had no negative or positive effect on trafficking to the plasma membrane relative to CHO cells transfected with only the melanocortin receptor. “Facilitates” indicates that the receptor did not translocate to the plasma membrane in the absence of the MRAP. “Restricts” indicates that there was a decline in trafficking to the plasma membrane when the receptor was co-expressed with an MRAP. For activation experiments, “not required” indicates that co-expression with an MRAP had no negative or positive effect on activation relative to CHO cells transfected with only the melanocortin receptor. “Requires” indicates that the receptor could not be activated when expressed alone in CHO cells. “Lowers” indicates that there was a statistically significant drop in activation when the receptor was co-expressed with an MRAP*.

**Figure 1:**
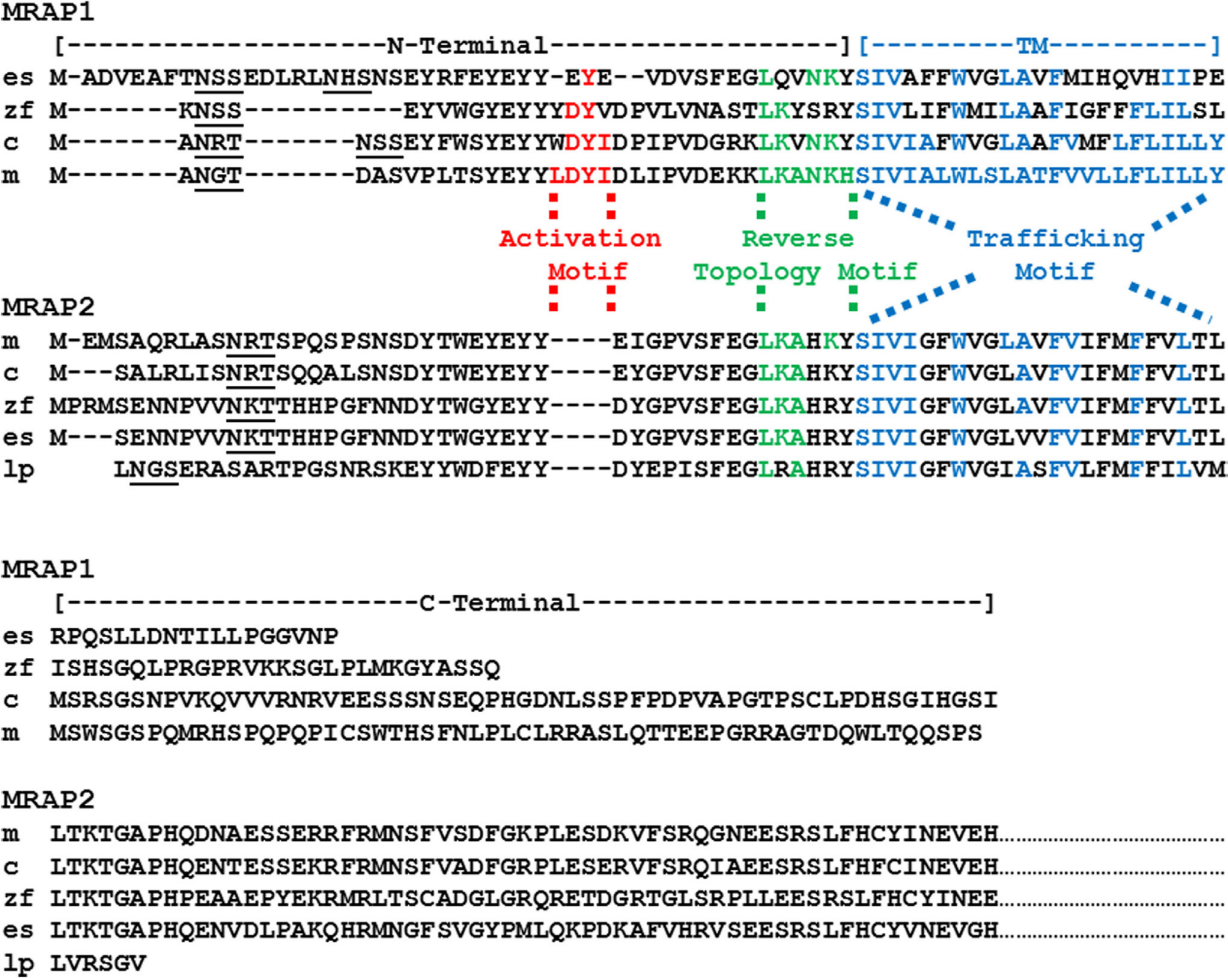
**Amino acid sequence alignment of MRAP1 and MRAP2 paralogs**. The amino acid sequences of mouse (m) MRAP1 (NP_084120.1), zebrafish (z) MRAP1 (XP001342923.2), chicken (c) MRAP1 (XR_001470382) elephant shark (es) MRAP1 (XM_007903550.1), mouse (m) MRAP2 (XP_006511239.1), chicken (c) MRAP2 (XP_015140201), zebrafish (zf) MRAP2a (XP_001342923.4), elephant shark (es) MRAP2 (XP_007906624.1), and lamprey (lp) MRAP2 (FAA00710.1) were aligned to the sequences of mouse MRAP1 and mouse MRAP2, respectively. Note that only a partial sequence for lamprey (*Petromyzon marinus*) MRAP2 has been reported (1). In addition, only the partial C-terminal sequences for the MRAP2 orthologs are presented. Predicted N-linked glycosylation sites are underlined. Note that there are two potential N-linked glycosylation sites in the putative elephant shark MRAP1 amino acid sequence. The amino acids in the activation motif for mouse MRAP1 are highlighted in red. Conserved amino acid positions in the proposed activation motifs of the chicken, zebrafish, elephant shark MRAP1 orthologs are also highlighted in red. The amino acids in the reverse topology motif of mouse MRAP1 were highlighted in green. Conserved amino acid positions in the reverse topology motif of chicken, zebrafish, elephant shark, mouse, and lamprey MRAP1 and MRAP2 sequences, respectively, were also highlighted in green. Finally, the amino acids in the transmembrane domain of mouse MRAP1 are highlighted in blue, and the conserved amino acid positions in the chicken, zebrafish, elephant shark, mouse and lamprey MRAP1 and MRAP2 sequences, respectively, were also highlighted in blue.

The salient features of the MRAPs are illustrated by mouse MRAP1 and MRAP2 (Figure 1). For MRAP1, the LKANKH motif is required for reverse topology (18), and the corresponding reverse topology motif in mouse MRAP2 is LKAHKY, [(8); Figure 1]. Reverse topology motifs are also apparent in the chicken and zebrafish MRAP1 and MRAP2 orthologs (Figure 1).

The TM domain of mouse Mrap1 is required for the trafficking of MC2R to the plasma membrane (18), and the corresponding sequence in mouse Mrap2 (Figure 1) has 43% amino acid sequence identity with the TM domain of mouse Mrap1. Among the MRAP1 orthologs presented in Figure 1, the amino acid sequence identity between mouse Mrap1 and chicken MRAP1, and mouse Mrap1 and zebrafish MRAP1 is 74 and 48%, respectively. This level of sequence identity is apparently adequate since both chicken MC2R (19) and zebrafish MC2R (20) can be activated when co-expressed in heterologous mammalian cells with their respective MRAP1 ortholog. It is interesting that the amino acid sequence identity for the TM region of the MRAP2 orthologs presented in Figure 1 is 74%. Since MRAP2 is expressed in brain and adrenal cortex cells, there appears to be selection pressure to maintain the TM sequences of MRAP2 orthologs. The physiological roles of the MRAP2 orthologs will be discussed later in this section.

Given the preceding comments on primary sequence similarity, the most striking difference between mouse Mrap1 and Mrap2 is the activation motif present in Mrap1 that is conspicuously absent in Mrap2 (Figure 1). As a result, although MC2R will move to the plasma membrane in the presence of MRAP2, activation of the receptor following an ACTH-binding event is barely detectable at concentrations of ACTH of 10^−8^M or less (8, 9, 21). Conversely, in the presence of MRAP1, the activation of MC2R is robust following an ACTH-binding event [(16); Table 1]. It would appear then, that when a mammalian MC2R ortholog is expressed alone, the receptor miss-folds, and is non-functional (22). When co-expressed with MRAP1, MC2R assumes an active conformation, and the MC2R/MRAP1 complex can be activated by ACTH. Interaction with an MRAP1 paralog to achieve functional expression is a strict requirement for teleost and tetrapod MC2R orthologs (20, 23).

Sebag and Hinkle (18) found that if the activation motif (LDYI) in the N-terminal domain of mouse Mrap1 (Figure 1) was replaced with alanine residues, the activation of MC2R was blocked. Furthermore, a single alanine substitution of the Y residue in the mouse Mrap1 LDYI motif resulted in a 50% drop in activation. Note that similar activation motifs are present in the N-terminal domains of the chicken and zebrafish MRAP1 orthologs (Figure 1). In addition, a recent study indicated that alanine substitution of the DY residues in the YDYV motif of zebrafish MRAP1 blocks activation of MC2R (24).

While it appears that the direct interaction of mammalian MRAP2 orthologs with mammalian MC2R orthologs might be pharmacological rather physiological [Table 1; (8, 9, 21)], there are cells such as mouse adipocytes (25) and embryonic mouse adrenal cortex cells (26) that co-express Mrap2 as well as Mc2r and Mc5r. Sebag and Hinkle (27) found that when human MC5R and mouse Mrap2 were co-expressed in CHO cells, the trafficking of human MC5R to the plasma membrane decreased. The implication of these experiments was that by decreasing the number of MC5 receptors on the plasma membrane, Mrap2 would make the target cells more selective for stimulation by ACTH, rather than α-MSH. Since, MC2R and MC5R are co-expressed in chicken adrenal cortex cells (28), frog interrenal tissue (23), and rainbow trout interrenal tissue (29), an interaction between MC5R and MRAP2 could have physiological implications for non-mammalian vertebrates as well. Hence, an evaluation of the pharmacological interactions of MC5R and MRAP2 orthologs with respect to trafficking to the plasma membrane and ligand selectivity in these species is warranted.

When considering a physiological role for MRAP2, a promising area of study has been the interaction between MRAP2 and MC4R in the modulation of feeding behavior by neurons in the hypothalamus (30). In both zebrafish (31) and mice (32), endogenous MRAP2 orthologs appear to play roles in the ligand sensitivity of the MC4 receptor. For example, Asai et al. (32) observed that when mouse Mc4r and Mrap2 are co-expressed in HEK-293 cells, Mc4R has a higher sensitivity for α-MSH; an outcome that would decrease feeding activity *in vivo*. Conversely, in mice in which the *Mrap2* gene was selectivity deleted from neurons in the hypothalamus, the result was an obese phenotype. While the implications of these results are intriguing from a biomedical perspective, it appears that for mammals there may be species specific difference in the regulation of MC4R. For example, Kay et al. (33) observed that co-expression of human MC4R and human MRAP2 in HEK-293 cells had no effect on ligand sensitivity. However, a shift in ligand sensitivity was observed when human MC4R was co-expressed with human MRAPα. Clearly, studies are needed on other tetrapods (i.e., amphibians, reptiles, and birds) are needed to determine the role that MRAP2 may play in modulating the ligand selectivity of MC4R in the regulation of feeding behavior in these organisms.

A more complex mechanism for the role of MRAP2 in regulating feeding behavior has been observed for the zebrafish (31, 34). As a result of a teleost-specific genome duplication [3R event; (35)], two paralogs of the *mrap2* gene (*mrap2a* and *mrap2b*) are present in the zebrafish genome (20). Furthermore, the expression of these paralogs appears to be developmentally regulated. Sebag et al. (31) report that during the larval stage of development, MRAP2a lowers the ligand sensitivity of zebrafish MC4R (as measured by *V*max), and as a result the animal eats more; an outcome that would favor growth. During larval development, the expression levels of the zebrafish *mrap2b* gene are low. Conversely in the adult stage, zebrafish *mrapb* gene expression is elevated, and zebrafish *mrapa* gene expression declines. Sebag et al. (31) also report that co-expression of zebrafish MC2R and zebrafish MRAP2 in HEK-293 cells increases the sensitivity of zebrafish MC4R for α-MSH. These results are interpreted as giving the adult zebrafish fine control over food consumption; a trait that would be considered adaptive (31). Hence, it would appear that zebrafish MRAPb is functioning in a manner analogous to mouse Mrap2 (32). However, Aguilleiro et al. (34) observed that co-expression of zebrafish MC4R and zebrafish MRAP2a in HEK-293 cells resulted in a higher sensitivity of the zebrafish MC4R for ACTH as compared to α-MSH. The latter study proposes that ACTH may be playing a role in the control of feeding behavior, and that role can be influenced by the expression levels of zebrafish MRAP2a. In the later study, the *in vitro* effect of zebrafish MRAP2b on zebrafish MC4R ligand selectivity was not apparent. Finally, zebrafish MRAP2a had no negative or positive effect on the trafficking of zebrafish MC4R to the plasma membrane (31, 34); whereas zebrafish MRAP2b appeared to increase the surface expression of zebrafish MC4R (31). Hence, there appears to be species-specific differences in way the MC4R orthologs respond to interaction with MRAP2 (See Table 1) That said, at the molecular level it is not clear which domain(s) of MRAP2 (MRAP2a or MRAP2b) are making contact with MC4R to alter ligand selectivity. The possibility of an “activation motif” in MRAP2 orthologs, analogous to the activation motif in MRAP1 orthologs, has not been investigated.

In terms of the evolution of the MRAP gene family, an earlier review concluded that MRAP2 was the ancestral “MRAP” (1). This conclusion was based on the apparent absence of MRAP1 orthologs in the genomes of a cartilaginous fish (*Callorhinchus milii*, the elephant shark) and the lamprey (*Petromyzon marinus*), and the presence of MRAP2 orthologs in both these species. In this scenario, the duplication of the ancestral *mrap* gene may have occurred in the bony fishes following the divergence of the ancestral cartilaginous fishes and the ancestral bony fishes (36) over 420 million years ago. However, recent annotation of the elephant shark genome project^1^ revealed a cDNA (accession number: XM_007903550.1) that Blast analysis^2^ has identified as an MRAP1 ortholog. The deduced amino acid sequence of the putative elephant shark MRAP1 ortholog is presented in Figure 1. The putative elephant shark MRAP1 has a reverse topology motif (LQVNKY), and the TM region has 39% amino acid sequence identity with the mouse MRAP1 TM region. The C-terminal domain of the putative elephant shark MRAP1 is very short relative to the other MRAP1 orthologs. However, Sebag and Hinkle (18) have shown that the C-terminal of mouse MRAP1 is not required for either trafficking or activation of mammalian MC2R orthologs. The N-terminal domain of the putative elephant shark MRAP1 is nearly 43% longer than the other MRAP1 orthologs in Figure 1. By inserting gaps, it was possible to align these sequences and identify a putative activation motif (EYE) in the putative elephant shark MRAP1. The presence of the Y residue in this domain is particularly interesting, given the importance of this residue for mammalian and teleost MRAP1 orthologs (18, 24).

**Figure 2:**
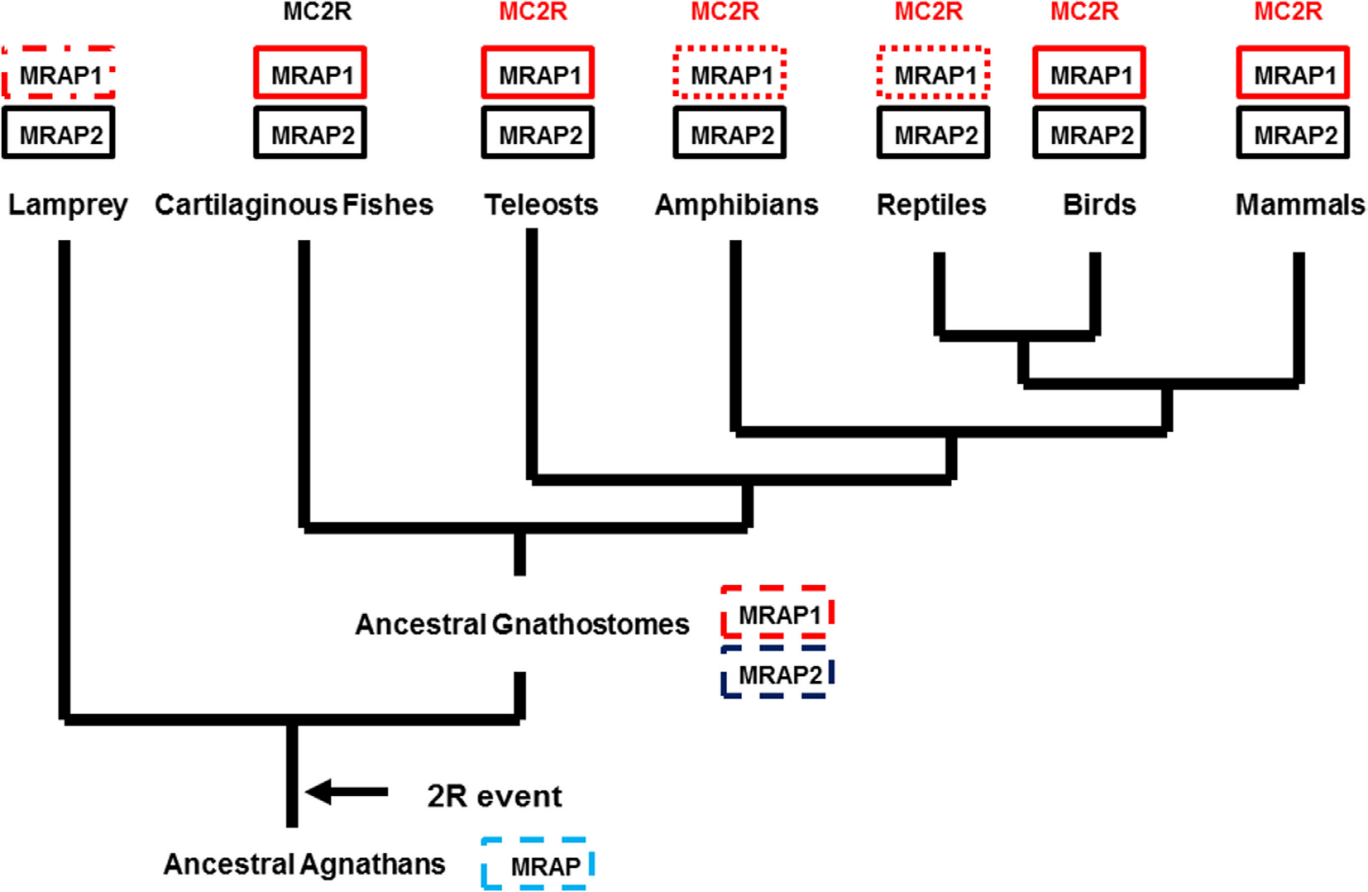
**Proposed evolution for the MRAP Gene Family**. The evolutionary tree presented in this figure assumes that there was an ancestral *mrap* (MRAP) gene in the genome of ancestral agnathans. Following the 2R genome duplication event, two paralogous mrap genes emerged (MRAP1 and MRAP2) and are present in the genomes of many extant 2R chordates. A solid black box indicates that the gene has been reported in the respective taxa. A box with a dashed line border indicates a gene that is predicted, but has not been detected (i.e., lamprey MRAP1) or the organism is extinct (e.g., ancestral agnathans, ancestral in gnathostomes). A box with a dotted border indicates a taxonomic group in which MRAP1 is required for the functional expression of MC2R, but a MRAP1 sequence has not been identified in the genome of a representative from that taxonomic group.

From a phylogenetic/evolutionary perspective, the detection of the putative cartilaginous fish MRAP1 ortholog fills a gap. The elephant shark is in Subclass Holocephali (Class Chondrichithyes), and it is very probable that *mrap1* orthologs are present in the genomes of members of Subclass Elasmobranchii (i.e., sharks and rays). Hence, *mrap1* and *mrap2* paralogs may have been present in the genome of the ancestral gnathostomes (Figure 2). Given these assumptions, the evolution of the *mrap* gene family may have involved the following scenario. In the ancestral agnathan vertebrates that underwent the 2R genome duplication event, the ancestral *mrap* gene would have been duplicated to yield the *mrap1* and *mrap2* genes, and these paralogous genes presumably would have been distributed on separate chromosomes. Currently, *mrap1* and *mrap2* genes have been found on separate chromosomes in the various gnathostome genome databases where chromosomes maps are available.^3^ Among extant 2R vertebrates (Figure 2), an *mrap1* ortholog has not been detected in the current version of the lamprey genome project^4^ (Figure 2). Whether the absence of this ortholog represents the incomplete state of the lamprey genome project, or a secondary loss of the ortholog cannot be determined at this time. In addition, *mrap1* orthologs have not been detected in the genomes of either the frog, *Xenopus tropicalis* or the reptile, *Anolis carolinensis*. However, the MC2R orthologs for both species requires co-expression with a tetrapod MRAP1 ortholog for functional expression in CHO cells (21, 37). It would appear that either the *X. tropicalis* and *A. carolinensis* genome projects are not complete, or some other accessory protein is utilized in these species.

From a pharmacological perspective, the presence of the putative elephant shark MRAP1 ortholog is perplexing. An earlier study had shown that the elephant shark MC2R ortholog could be functionally expressed in CHO cells in the absence of co-transfection of an exogenous *mrap1* cDNA (38). Elephant shark MC2R could be stimulated in a dose-dependent manner by either human ACTH (1–24) or by dogfish (*Squalus acanthias*) ACTH (1–25). More recently, a MC2R cDNA cloned from the genome of the stingray, *Dasyatis akajei*, was also functionally expressed in CHO cells in the absence of co-transfection of an exogenous *mrap1* cDNA (39). The stingray MC2R ortholog also could be stimulated by stingray ACTH (1–24) and stingray Des-Acetyl-α-MSH. Hence, there are several issues with respective to the putative elephant shark MRAP1 that need to be resolved. It will be important to determine whether the elephant shark *mrap1* mRNA is expressed in the same cells as elephant shark *mcr* mRNAs. In addition, pharmacological studies are needed to determine whether co-expression of cartilaginous fish MCR orthologs with the putative elephant shark MRAP1 ortholog have any effect on either trafficking of the MCR orthologs to the plasma membrane or sensitivity to melanocortin ligands.

## Ligand Selectivity of MC2R Orthologs

Several studies have shown that the MC2R orthologs of teleosts and tetrapods (Figure 2) require co-expression with a corresponding MRAP1 ortholog. Perhaps as a result of this interaction, and the intrinsic tertiary features of these MC2R orthologs, all of these receptors can only be activated by ACTH, and not by any MSH-sized ligand (19–21, 37, 40). Nearly 40 years ago, analog studies on mammalian ACTH sequences revealed the dual importance of the H^6^F^7^R^8^W^9^ motif and the K^15^K^16^R^17^R^18^ (tetrabasic) motif in ACTH for the activation of the “ACTH” (MC2R) receptor on mammalian adrenal cortex cells (41). These same features are required for the activation of MC2R on the interrenal and adrenal cortex cells of non-mammalian tetrapods and teleosts as well (42). Although, teleost and tetrapod α-MSH sequences have the H^6^F^7^R^8^W^9^ motif, these ligands lack the tetrabasic motif, and as a result are incapable of activating either teleost or tetrapod MC2R orthologs. By contrast, the teleost and tetrapod MC1R, MC3R, MC4R, and MC5R paralogs can be activated by either ACTH or the MSH-sized polypeptides derived from POMC with varying potencies (10, 42, 43). It would appear that teleost and tetrapod MCR paralogs all have an HFRW-binding site, and MC2R orthologs have an addition R/KKRR-binding site. These generalizations apply for the ligand selectivity properties of the MCRs of cartilaginous fishes (class Chondrichthyes) with one notable exception.

Studies on the ligand selectivity of dogfish, *Squalus acanthias* (order Squaliformes, subclass Elasmobranchii), MC3R, MC4R, and MC5R paralogs (44–46), and the MC1R, MC3R, MC4R, and MC5R paralogs of the stingray, *D. akajei* [order Rajiformes, subclass Elasmobranchii; (39)] found that these MCR paralogs could be activated by either ACTH or MSH-sized ligands in a manner analogous to the corresponding MCR paralogs in teleosts or tetrapods. Hence, these paralogs have an HFRW-binding site. However, the MC2R ortholog of the stingray, *D. akajei*, and the MC2R ortholog from the elephant shark, *C. milii* (order Chimaeriformes, subclass Holocephali) could also be activated by either ACTH or MSH-sized ligands (38, 39), and as noted in Phylogeny and Proposed Evolution of the MRAPs, both of these MC2R orthologs could be functionally expressed in CHO cells without co-expression of an exogenous *mrap1* cDNA. The two cartilaginous fish MC2R orthologs, from different subclasses of the cartilaginous fishes, have ligand selectivity properties more similar to MC4R paralogs, and most likely have only a HFRW-binding pocket. This apparent feature for the cartilaginous fishes MC2R orthologs would be quite distinct from teleost and tetrapod MC2R orthologs. Whether the ligand selectivity properties of the cartilaginous fishes MC2R orthologs are an ancestral trait or a derived trait unique to the cartilaginous fishes is not clear at this time.

**Figure 3:**
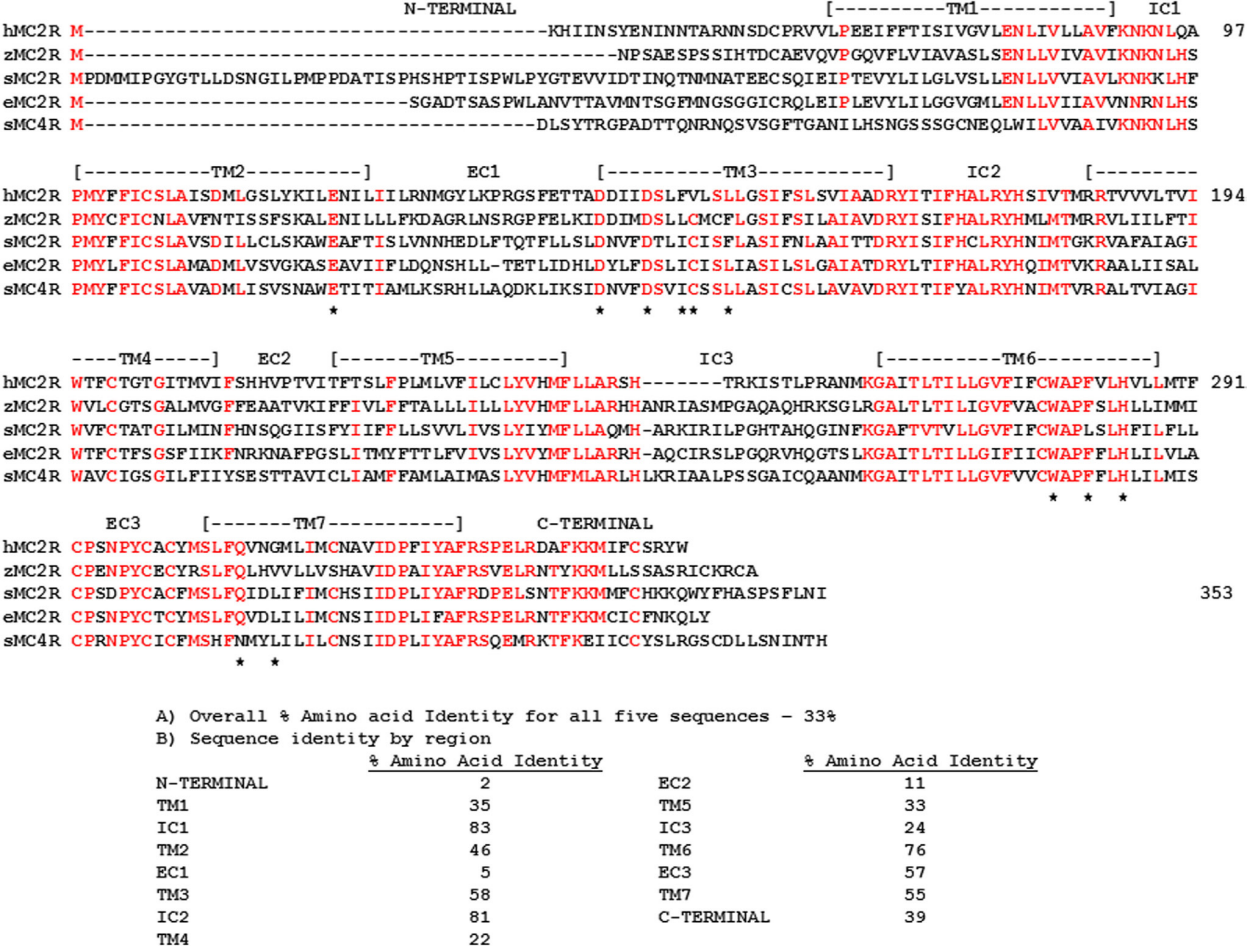
**Amino Acid Alignment of MC2R Orthologs**. The amino acid sequences of human (h) MC2R (NP_ 001278840.1), zebrafish (z) MC2R (XP_00518229.1) stingray (s) MC2R (LC108747), elephant shark (e) MC2R (FAA704.1), stingray (s) MC4R (LC108749) were aligned, and amino acid positions in which four of the five sequences were identical are marked in red. The position of critical amino acids in the HFRW-binding site of MC4R orthologs (47) are marked with a star. The overall percent primary sequence identify **(A)** and the percent identity within each domain **(B)** are presented.

In any event, there should be distinct sites within the teleost/tetrapod MC2R orthologs and the cartilaginous fishes MC2R orthologs that can account for the ligand selectivity properties of these receptors. In this regard, a comparison of MC2R orthologs with an MC4R paralog my reveal these potential sites. As shown in Figure 3, the human, zebrafish, elephant shark, and stingray MC2R amino acid sequences could be aligned to the stingray MC4R sequence by inserting a minimum of two gaps. The positions of critical residues in TM2, TM3, TM6, and TM7 that correspond to the HFRW-binding site for a MC4R ortholog (47) are marked with a star. For the teleost and tetrapod MC2R orthologs, six of these sites are conserved, and for the cartilaginous fishes MC2R orthologs eight of these positions are conserved. Overall only 33% of the positions in this alignment are identical in at least four of the five sequences, however, there are very clear regions of primary sequence identity, which serve as markers for MCR-related sequences. The highly conserved regions (sequence identify greater than 50%) include: IC1, TM3, IC2, TM6, EC3, and TM7. Moderately conserved regions (sequence identity greater than 35%) include: TM1, TM2, and the C-terminal domain. The highest primary sequence divergence (<15%) was observed for the N-terminal domain, EC1, and EC2.

Previous studies used chimeric proteins of human MC2R and human MC4R to analyze the functions of these regions. For example, Fridmanis et al. (48) observed that replacing the N-terminal domain of human MC4R with the N-terminal domain of human MC2R inhibited trafficking of the chimeric MC4R protein to the plasma membrane. However, since the N-terminal of stingray MC4R is nearly the same length as the human MC2R domain (Figure 3), and the stingray receptor could be functionally expressed in CHO cells (39), length alone may not be a factor in influencing trafficking to the plasma membrane. Hinkle et al. (49) observed that exchanging the TM2/EC1/TM3 region of human MC2R with the corresponding region of human MC4R resulted in a chimeric MC2R protein that could be activated by either ACTH or NDP-MSH. Presumably making a similar chimeric protein for human MC2R, but using the TM2/EC1/TM3 region of either elephant shark or stingray MC2R should yield the same outcome. Finally, Fridmanis et al. (48) observed that substitution of the TM4 and TM5 domains in human MC2R affected ACTH activation, and this region of human MC2R may be the KKRR-binding site. In support of the later conclusion, studies on naturally occurring mutations in the TM4/EC2/TM5 domain of human MC2R, and alanine substitution experiments point to the TM4/EC2/TM5 region as playing an important role in the activation of human MC2R [for review see Ref. (50)]. It would now seem advantageous to extend the chimeric protein paradigm to the cartilaginous fishes MC2R orthologs to determine whether exchanging the TM2/EC1/TM3 and TM4/EC2/TM5 domains of teleost/tetrapod MC2R orthologs with the corresponding domains in the elephant shark MC2R orthologs would make the cartilaginous fish MC2R chimeric proteins exclusively selective for ACTH, and in the converse experiments, would the teleost/tetrapod chimeric MC2R proteins have more permissive ligand selectivity properties. Given Malik et al. (51) observations on the importance of extracellular domains in human MC2R for interaction with mouse MRAP1, co-expression of these MC2R chimeric proteins with a class-specific MRAP1 ortholog may reveal the domain within the MC2R orthologs that makes contact with MRAP1.

## Melanocortin Receptor Genome and Gene Duplications

**Figure 4:**
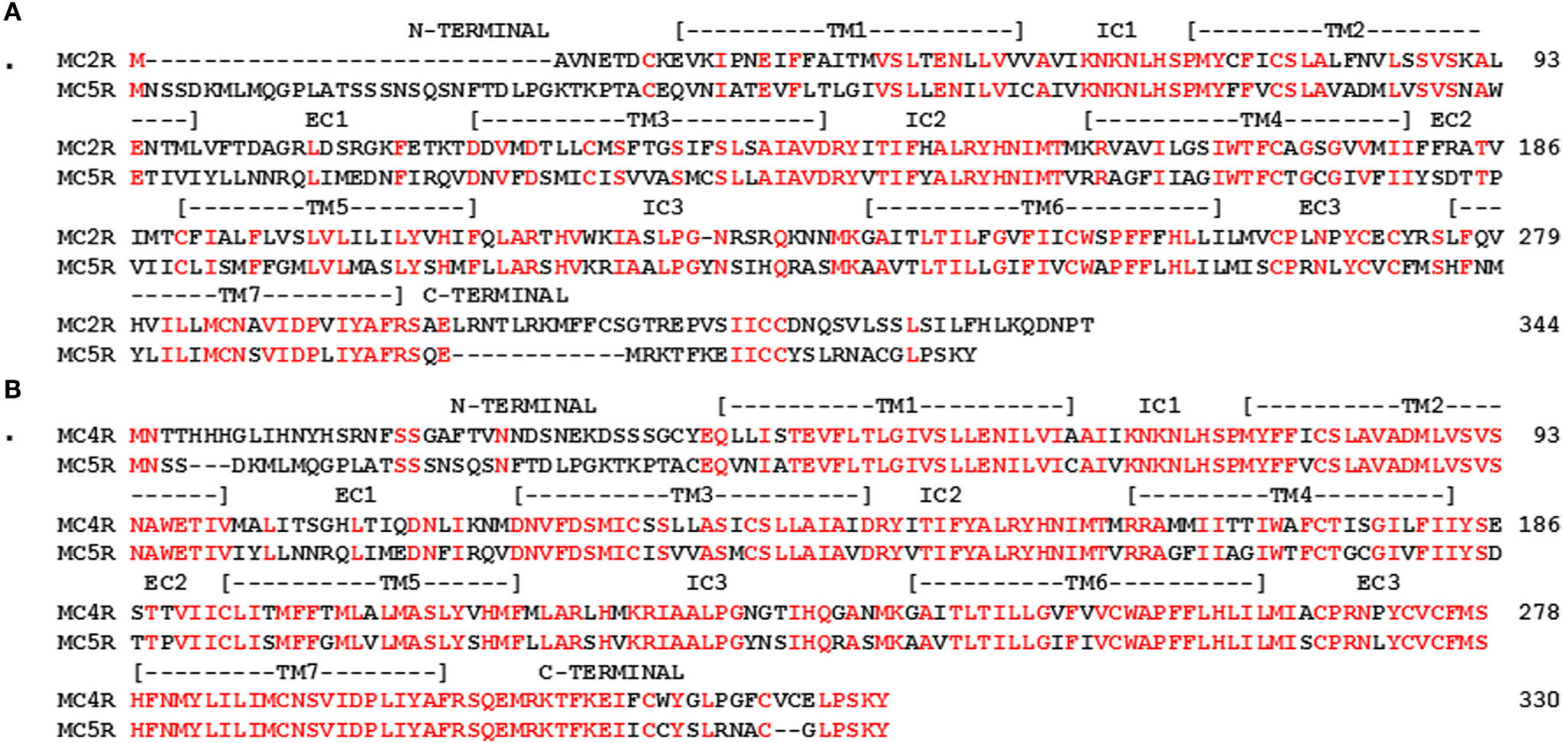
**Amino Acid alignment of Gar MC2R, MC4R, and MC5R**. The amino acid sequences of gar MC2R (ENSLOCT00000011667), gar MC4R (ENSLOCT00000022303), and gar MC5R (ENSLOCG00000018340) were aligned and positions that were identical are marked in red. **(A)** alignment of gar MC2R and gar MC5R; **(B)** alignment of gar MC4R and gar MC5R.

The successive genome duplications during the radiation of the chordates theoretically should yield four paralogous genes in the genomes of extant cartilaginous fishes, non-teleost ray finned fishes, and tetrapods. However, in the genomes of the Japanese stingray (39), the spotted gar^5^, or the mouse (6), there are five paralogous MCR genes. The conclusion drawn from these observations is that one of the paralogous *mcr* genes underwent a local gene duplication (52). While there is general agreement that the *mc5r* gene was the result of the local gene duplication, the original *mcr* paralog that was duplicated has not been resolved. The issues associated with the origin of the *mc5r* gene can be seen in the spotted gar (sg) genome. Chromosome mapping indicates that the *sgmc1r* gene is located on chromosome 21, the *sgmc2r* gene is located on chromosome 11, the *sgmc3r* gene is located on chromosome 18, the *sgmc4r* gene is located on chromosome 9, and the *sgmc5r* gene is also located on chromosome 11. The presence of paralogous genes on different chromosomes is considered an indication of a genome duplication event(s) (4). The presence of two genes on the same chromosome is generally construed as a result of a local gene duplication. In this regard, synteny studies found that the *mc2r* and the *mc5r* genes were on the same chromosome in the genomes of teleost fishes, the chicken (*Gallus gallus*), and several mammals (43, 52, 53). Based on these observations, it seemed reasonable to conclude that the *mc2r* and *mc5r* genes were the result of a local gene duplication (54, 55). In this scenario, the paralogous *mc2r* and *mc5r* genes would accumulate mutations independently, and based on selection pressures diverge in terms of amino acid sequence, and perhaps in terms of function. The divergence in amino acid sequence can be seen from an alignment of gar MC2R and gar MC5R (Figure 4A). The amino acid identity for the two paralogs is 42%. From an evolutionary perspective, the local gene duplication may have occurred after the 2R genome duplication event in the ancestral gnathostomes prior to the divergence of the ancestral cartilaginous fishes and the ancestral bony fishes.

However, Vastermark and Schioth (1) have pointed out that in phylogenetic analyses, MC5R orthologs form a clade with MC4R orthologs, and do not form a clade with MC2R orthologs. These observations have led to the conclusion that the *mc5r* gene was the result of a duplication of the *mc4r* gene (1). In support of this conclusion, an alignment of gar MC4R and gar MC5R indicates 70% sequence identity (Figure 4B). In this scenario, the presence of the *mc2r* and *mc5r* paralogous genes on the same chromosome of extant teleost and tetrapods could have been the result of an exchange of chromosome fragments in the last common ancestor to the ancestral ray-finned fishes (Class Actinopterygii) and the ancestral lobe-finned fishes (Class Sarcopterygii), the lineage which gave rise to the tetrapods, approximately 410 million years ago (56).

**Figure 5:**
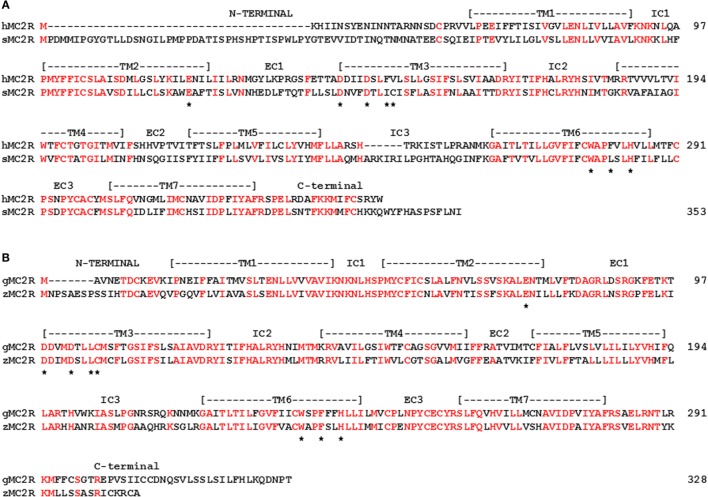
**Amino acid sequence identity of MC2R orthologs**. **(A)** The amino acid sequences of human (h) MC2R and stingray (s) MC2R were aligned. **(B)** The amino acid sequences of gar (g) MC2R and zebrafish (z) MC2R (XP_005158229) were aligned. The position of critical amino acids in the proposed HFRW-binding site (47) are marked with a star. Amino acid positions that are identical are marked in red.

While either scenario (i.e., MC2R/MC5R or MC4R/MC5R) can be supported by the current evidence, there are at least two issues that neither scenario adequately addresses. As shown in Figure 5A, a comparison of stingray MC2R and human MC2R, vertebrates that last shared a common ancestor over 420 million years ago, the amino acid sequence identity is 37% (positions in red). Given the apparent role of the Hypothalamus/Pituitary/Adrenal (HPA) axis and the Hypothalamus/Pituitary/Interrenal (HPI) axis in maintaining the fitness of vertebrates (57–60), this lack of primary sequence conservation is difficult to comprehend. While divergence is expected, this degree of divergence is difficult to rationalize. It would appear that during the radiation of the gnathostomes, the *mc2r* gene sequence has drifted to the current state, while still maintaining functional capabilities.

For ray-finned fishes, such as the gar or zebrafish (Class Actinopterygii), MC2R primary sequence conservation is higher (55%; Figure 5B), and this condition may reflect the close interaction with MRAP1. That interaction most likely started in the ancestral bony fishes, and while the interaction may not have “rescued” MC2R functionality, the interaction appears to have stabilized the functional capabilities of teleost and tetrapod MC2R orthologs. Tetrapod MC2R orthologs show a similar level of primary sequence conservation. As a result, selection pressures on teleost and tetrapod MC2R orthologs may involve maintaining the close interaction between MRAP1 and MC2R. For the cartilaginous fishes, the MRAP1/MC2R relationship is unclear or may not exit, and the selection pressures to maintain MC2R primary sequence identity does not appear to be as strong. For example, in a recent study, on stingray MCRs (39), *mc2r* and *mc5r* mRNA levels were detected in the interrenal tissue of this species. However, the EC_50_ value of the stingray MC5R for ACTH (1–24) was in the 10^−9^M range, whereas the EC_50_ value for stingray MC2R was in the 10^−7^M range. In this example, MC5R rather MC2R may be the “ACTH” receptor in the HPI axis of the stingray.

**Figure 6:**
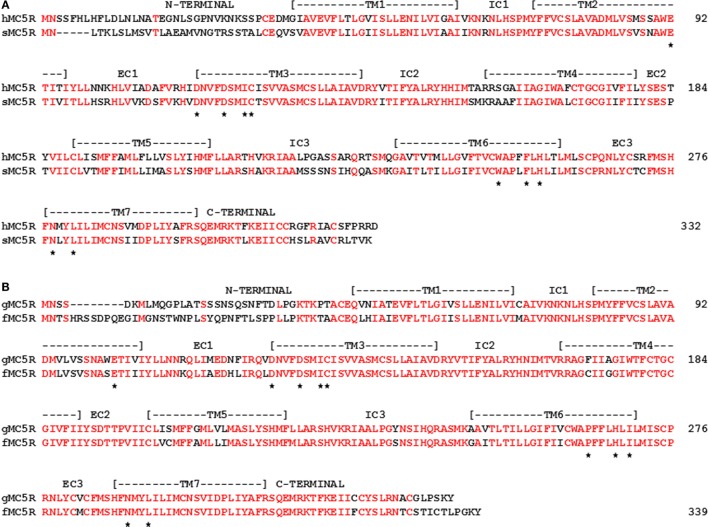
**Amino Acid Sequence Identity of MC5R Orthologs**. **(A)** The amino acid sequences of human (h) MC5R (NP_ 005904.1) and stingray (s) MC5R (AY562212) are aligned. **(B)** The amino acid sequences of gar (g) MC5R and *Takifugu rubripes* (f) MC5R (AA06553.1; fugu) were aligned. The position of critical amino acids in the proposed HFRW-binding site (47) is marked with a star. Amino acid positions that are identical are marked in red.

The diminished primary sequence conservation for MC2R orthologs is in sharp contrast to the higher degree of primary sequence conservation for stingray and human MC5R orthologs (55%; Figure 6A). In addition, for two very distantly related bony fish MC5R orthologs (gar and fugu) the sequence identity was 73% (Figure 6B). These observations beg the question of the functional significance of the stability of MC5R orthologs during the radiation of the gnathostomes. For mammals, MC5R plays a role in exocrine gland secretion (61). However, the role of the MC5R receptor in non-mammalian vertebrates is largely unknown. Perhaps a renewed focus on the distribution of MC5 receptors in various tissues of non-mammalian vertebrates will yield some answers.

## Conclusion

While it has been nearly 25 years since the cloning of the first MCRs (6, 11), and nearly 40 years since the structure/function studies on ACTH (41), there are still many aspects of the pharmacology and physiology of the melanocortin peptides and the MCRs that have not been resolved. One of the interesting facets of this receptor family is the interaction with the MRAPs (8, 9, 16, 17). The presence of a *mrap1* ortholog in the genome of a cartilaginous fish suggests that the *mrap1* and the *mrap2* paralogous genes were the result of the 2R genome duplication event (2). There is still a considerable amount of work to be done to clarify the physiological roles of the MRAP1 and MRAP2 in non-mammalian vertebrates, and the contact sites between MRAPs and the MCRs that can influence ligand selectivity.

When considering the functional activation of the MCRs, the paralogs MC1R, MC3R, MC4R, and MC5R are activated through an HFRW-binding site on these receptors that appears to be highly conserved. However, the MC2R orthologs of teleosts and tetrapods appear to utilize an additional binding site for the R/KKRR motif in gnathostome ACTH. Recent studies on cartilaginous fish MC2R orthologs suggest that a single-binding site may be all that is needed for the activation of these receptors (39, 46). Identifying the functional domains within the various gnathostome MC2R orthologs may clarify the ligand selectivity properties of cartilaginous fish and teleost/tetrapod MC2R orthologs.

Finally, while the role for MC2R orthologs in the HPA/HPI axis seems very clear, the role of the MC5R orthologs in the physiology of non-mammalian vertebrates is not resolved. The possibility that MC2R and MC5R may be functioning in the same cells should be considered.

## Author Contributions

The author confirms being the sole contributor of this work and approved it for publication.

## Conflict of Interest Statement

The authors declare that the research was conducted in the absence of any commercial or financial relationships that could be construed as a potential conflict of interest.

## Footnotes

^1^http://esharkgenome.imcb.a-star.edu.sg

^2^https://blast.ncbi.nlm.nih.gov/Blast.cgi

^3^http://ensemble.org

^4^http://www.ensembl.org/Petromyzon_marinus/Info/Index

^5^http://www.ensembl.org/Lepisosteus_oculatus/Info/Index
